# Acute Invasive Fungal Rhinosinusitis-Related Orbital Infection: A Single Medical Center Experience

**DOI:** 10.1155/2021/9987871

**Published:** 2021-06-12

**Authors:** Yu-Fang Huang, Kai-Li Liang, Chiao-Ying Liang, Po-Chin Yang, Jun-Peng Chen, Li-Chen Wei

**Affiliations:** ^1^Department of Ophthalmology, Taichung Veterans General Hospital, Taichung, Taiwan; ^2^Department of Otolaryngology, Taichung Veterans General Hospital, Taichung, Taiwan; ^3^Center for Geriatrics and Gerontology, Taipei Veterans General Hospital, Taipei, Taiwan; ^4^Biostatistics Task Force of Taichung Veterans General Hospital, Taichung, Taiwan

## Abstract

**Backgrounds:**

Acute invasive fungal rhinosinusitis (AIFRS) is a hazardous infectious disease with rapid progression and high mortality and morbidities. Further orbital involvement is commonly seen. This study aims to analyze risk factors, clinical characteristics, and outcomes between patients with or without orbital involvement.

**Methods:**

A retrospective review was performed in a single tertiary medical center over a span of 13 years (2005–2018). A total of 21 patients with diagnosis of AIFRS were enrolled. We reviewed the patients' basic characteristics, comorbidities, clinical presentations, image study findings, culture pathogens, and treatment outcomes and analyzed the differences between orbital-involved and orbital sparing disease.

**Results:**

The most common comorbidities in AIFRS were diabetes mellitus (DM) and hematological malignancy. Nine the 21 AIFRS patients had orbital-involved disease. Patients with orbital involvement had a higher prevalence of DM (*p* < 0.05). Image studies revealed significant infection of the ethmoid sinus, sphenoid sinus, and frontal sinus in the group with orbital complication (*p* < 0.05). *Mucor*, *Rhizopus,* and *Aspergillus* were cultured in both groups. Five patients in the orbital involvement group expired, with all of them having an initial presentation of conscious disturbance (*p* < 0.01). Rhino-orbital-cerebral fungal infection was noticed in 3 of the 5 expired patients.

**Conclusion:**

In AIFRS patients, DM other than hematological malignancy was the main risk factor for orbital-involved disease. Patients with ethmoid, sphenoid, or frontal sinusitis had a higher possibility of orbital complication. Poor consciousness at initial presentation revealed highest possibility of rhino-orbital-cerebral fungal infection and led to death.

## 1. Introduction

Fungal rhinosinusitis is not uncommon and is typically classified into invasive and noninvasive disease. Invasive fungal rhinosinusitis is usually divided into acute and chronic, according to the clinical disease course. Acute invasive fungal rhinosinusitis (AIFRS) is a rare but life-threatening infectious disease. It demonstrates both a fulminant clinical course, as well as provides pathological evidence of angioinvasion by fungal hyphae or acute tissue necrosis [1–3]. AIFRS is an opportunistic disease seen mostly in immunocompromised patients. It sometimes progresses into an orbital fungal infection. Patients with orbital involvement would present with eyelid erythema, proptosis, ptosis, chemosis, limitation or pain on ocular movement, decreased vision, or abnormal light reflex [4]. Diagnosis is made with either a culture or pathologic report from nasal, palatal, or orbital tissue, as well as image studies (CT or MRI) which may provide information on any disease extension. Though the administration of antifungal agents and aggressive surgical debridement are provided upon diagnosis, fungal rhino-orbital cellulitis results in a high mortality and morbidities. In this study, we presented serial patients who were diagnosed with AIFRS in a single tertiary hospital and attempted to find differences between orbital-involved and orbital sparing disease. Due to rapid progression of AIFRS, the study is dedicated to analyzing risk factors and early detection of orbital or even cerebral involved disease to reduce mortality and improve prognosis.

## 2. Methods

We retrospectively reviewed the electronic medical records from 2005 to 2018 in Taichung Veterans General Hospital, Taiwan. Subjects who were diagnosed with AIFRS were enrolled according to the classification system proposed by deShazo et al. [5]. It was not appropriate or possible to involve patients or the public in the design, conduct, reporting, or dissemination plans of our research.

These AIFRS patients were presented with a fulminate infectious course of less than four weeks. All the patients showed either histopathological evidence of fungal hyphae invasion into vessels or acute tissue necrosis. Orbital involvement was defined as orbital fat stranding, subperiosteal abscess or extraocular muscle swelling seen on CT or MRI, or if the patient was presented with symptoms of orbital cellulitis, including proptosis, conjunctival chemosis, and ophthalmoplegia.

Data collected included age, gender, and any underlying systemic disease. Presenting symptoms and signs such as nasal, orbital, and facial involvement or central nervous system complaints were also recorded. Image studies such as CT, MRI, and culture reports, which included fungus and bacteria were documented. Also, the time from symptoms onset to diagnosis, medical and surgical treatment, and outcome were reviewed. The statistical analysis included the chi-square test or Fisher's exact test to compare factors such as comorbidities, presented symptoms and signs, culture pathogen, and image findings. The Mann–Whitney *U* test was used for comparing age and onset days. Analyses were performed using the Statistical Package for the Social Science (IBM SPSS version 22.0; International Business Machines Corp, New York, USA). A *p* value <0.05 was considered significant.

## 3. Results

During 2005 to 2018, twenty-two patients were diagnosed with AIFRS in Taichung Veterans General Hospital, Taiwan. One was excluded due to disseminated mucormycosis, in which we could not define the origin of the orbital or sinus infection. The remaining 21 patients were enrolled for analysis. Nine of the 21 patients had orbital involvement and were aged from 44 to 76 years, with a median age of 64. The group without orbital involvement included 12 patients, aged from 34 to 73 years, with a median age of 55. The median time for symptoms onset to diagnosis was 14 days in the orbital involvement group and 13 days in the orbital sparing group.

### 3.1. Systemic Disease and Predisposing Conditions

In the orbital involvement group, all patients had diabetes mellitus. In these 9 diabetic patients, seven of them had been checked with HbA1C level which ranged from 7.6% to 14.8% and 6 patients had HbA1C level higher than 9%. Six patients had hypertension, while 4 had chronic kidney disease. One patient had been diagnosed with Behcet's disease and was receiving immunosuppressants and oral steroids, while another was a case of acute myeloid leukemia.

Of the 12 patients with orbital sparing disease, malignancy was the most common underlying disease, with 2 having a solid tumor and 8 having hematological malignancy.

There were significant differences in the underlying diseases between the two groups. DM, hypertension, and renal insufficiency were risk factors for the orbital-involved disease group (*p* < 0.05), while patients without orbital complication had a higher prevalence of hematological malignancy (*p*=0.024).

### 3.2. Symptoms and Signs

**Figure 1:**
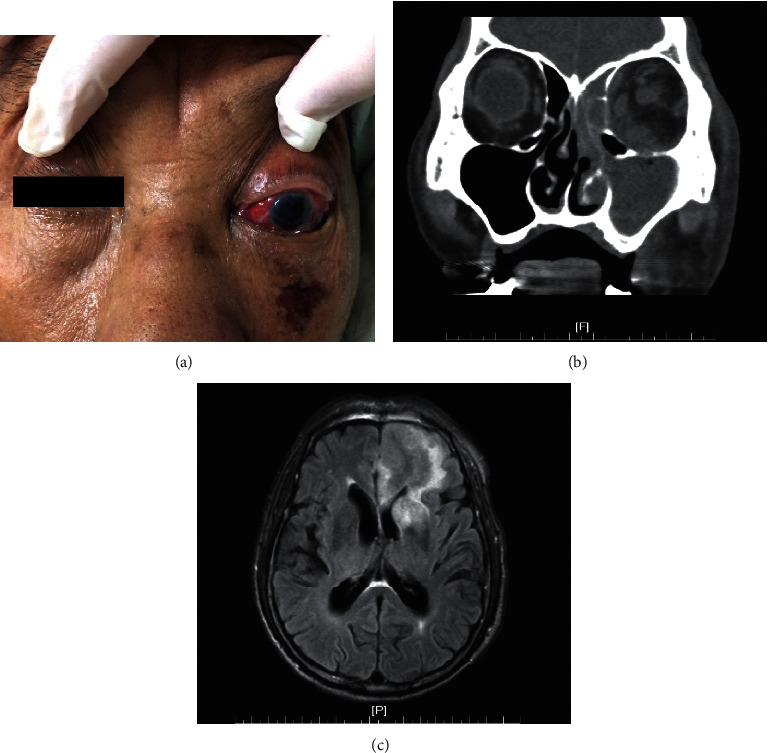
This was a 73-year-old male patient. He died 22 days after disease onset. The culture reported *Rhizopus*. (a) The patient presented as left facial swelling and tenderness, left eye proptosis, painful extraocular movement, eyelid swelling and erythema, conjunctival injection and chemosis, and cornea edema. (b) Computed tomography revealed orbital intraconal infiltration, extraocular muscles swelling, and sinusitis over maxillary and ethmoid sinuses. (c) Magnetic resonance imaging was performed 2 days after the CT, which revealed ipsilateral brain edema with midline shift.

**Figure 2:**
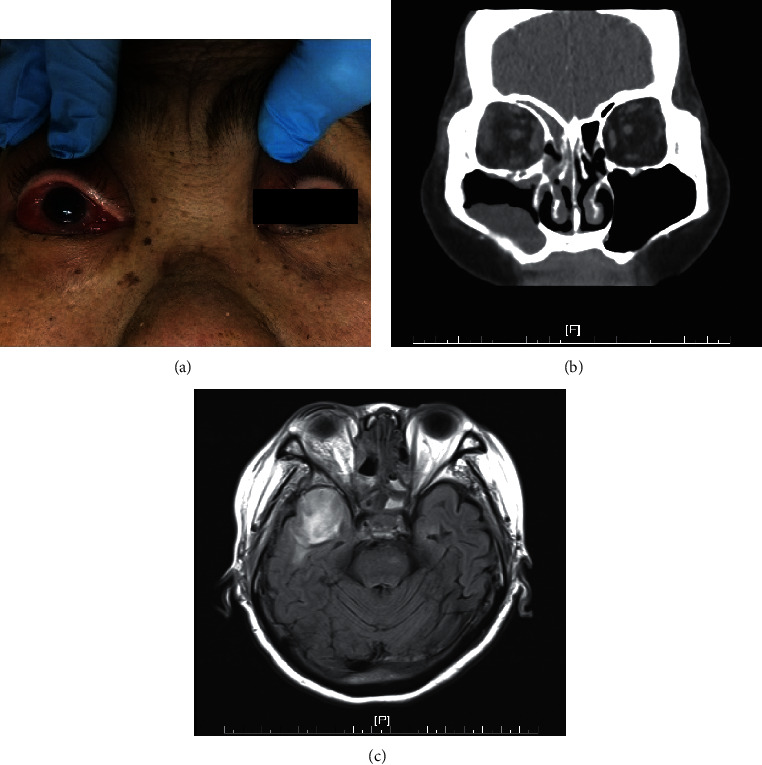
This was a 67-year-old female patient. Her pathological diagnosis was *Mucor* spp. She died 26 days after admission. (a) She presented as right eye blurry vision, limited extraocular movement, conjunctival injection and chemosis, and mild proptosis. (b) Computed tomography revealed orbital infiltration and subperiosteal abscess. Fluid accumulation over the frontal, ethmoid, and maxillary sinuses was seen. (c) Magnetic resonance imaging showed ipsilateral brain edema over the temporal region. This was taken 18 days after the CT image (b).

During the initial disease course, most patients in both groups did not present typical sinusitis symptoms such as cheek pain, headache, nasal obstruction, or nasal discharge. However, orbital pain, ophthalmoplegia, decreased visual acuity, and conscious disturbance were found to be significantly higher in the orbital involvement patients (*p* < 0.05), while fever was much more common in the no orbital involvement group (*p*=0.002). Ptosis, orbital swelling, and headache were noted in 4 of the 9 patients with orbital involvement. However, these symptoms showed no significant difference between the two groups. Figures 1 and 2 presented the symptoms and manifestations of 2 rhino-orbital-cerebral fungal infection patients.

### 3.3. Image Findings

Most patients received either a sinus or orbital CT, with some receiving an MRI. Sinus involvement was documented. In the orbital-involved group, all 9 patients received CT study and 3 had also undergone MRI examination. The image findings showed that ethmoid sinusitis consisted 100% patients, and 88.9% and 66.7% with sphenoid and frontal sinusitis, respectively, which were shown to be significantly high in the orbital involvement group (*p* < 0.05). In these 9 patients, eight of them had intraconal infiltration, six patients had extraocular muscles swelling, four had optic nerve swelling, and 5 showed subperiosteal abscess, and all these four image findings showed no differences between dead or alive patients. Three of 9 patients had brain abscess and brain edema on image study, and all had expired due to infectious disease. Detailed variables are listed in Tables 1 and 2.

### 3.4. Pathogens and Treatments

In 9 patients with orbital complication, a fungal culture reported *Rhizopus* in 4 patients, *Aspergillus* in 3 patients, and *Mucor* and *Rhizomucor* in 2 patients and 1 patient, respectively. One reported two species as *Rhizopus* and *Aspergillus.* Nine of 12 patients without orbital involvement reported *Aspergillus*. There were no significant differences in the fungal cultures taken between the two groups.

All patients received systemic antifungal treatment. Amphotericin B or liposomal amphotericin B was used in approximately 80% of the enrolled patients. Some patients shifted to posaconazole during admission, mainly due to the side effects caused by the original antifungal agents. Four patients with culture reported *Aspergillus* were treated with voriconazole. Patients with a bacterial coinfection were treated with antibiotics according to the minimum inhibitory concentration reports.

All patients in both groups, other than the two who had expired due to acute myeloid leukemia, received endoscopic sinus surgery (ESS) at least once. In the orbital involvement group, one was found with vitreous opacity and underwent evisceration to the affected eye due to the suspicion of fungal endophthalmitis. An additional patient who had a brain abscess was diagnosed with invasive aspergillosis and received craniotomy and abscess drainage. Another patient had orbital subperiosteal abscess seen on a follow-up CT image and underwent ESS and anterior orbitotomy for debridement. Finally, one patient who had panophthalmitis received 5 times intravitreous injections with amphotericin B because this patient's family refused further surgical management.

### 3.5. Outcome

In a total of 9 patients with orbital involvement, five died due to fungal infection, one of whose culture reported *Mucor* spp., while two patients separately reported *Aspergillus* and *Rhizopus.* Two of the 9 patients experienced full recovery without any sequelae, while one had profound visual disability and pthisis bulbi. One additional patient had the remaining symptom as mild nasal numbness.

In 12 patients without orbital involvement, seven expired due to their original hematological malignancy during admission. One patient had persistent right eye ptosis, and 2 had soft palate perforation. One patient was a case of adenoid cystic carcinoma who is still receiving cancer treatment. The remaining one patient was under stable condition.

Comparing the outcomes of the orbital involvement group, we found that all 5 patients who had died of infection initially presented as conscious disturbance, while none of the surviving patients had conscious change during hospitalization (*p* = 0.008). Three of the 5 expired patients had brain abscess or brain edema shown on their follow-up images. The disease onset days were longer in the expired patients, and also a higher percentage of patients reported orbital symptoms such as orbital pain, swelling, ptosis, and ophthalmoplegia, though all these factors revealed no significant statistical differences between dead or alive patients. The details are shown in Table 2.

## 4. Discussion

AIFRS is a rare disease and is almost only seen in immunocompromised patients. Fungal rhino-orbital cellulitis mostly occurs as a contiguous infection from rhinosinusitis, which is a devastating disease with a high mortality rate.

In this retrospective series, diabetes mellitus was seen in all patients with orbital involvement, while malignancy was noticed in most patients without orbital infection. Several retrospective analyses of rhino-orbito-cerebral mucormycosis or fungal orbital cellulitis have revealed a higher prevalence of diabetes mellitus, particularly diabetic ketoacidosis. Diabetic patients have an impaired immune function due to less chemotaxis and phagocytosis drawn by neutrophils, monocytes, and macrophages. An environment of low oxygen tension, hyperglycemia, and ketosis provides an excellent medium for the fungus to thrive [6–14]. Thus, uncontrolled DM was a predisposing risk for invasive fungal rhinosinusitis to progress.

In our analysis, patients with hematological malignancy experienced a much lower incidence of orbital infection. These patients were documented with neutropenia during hospitalization, and some died due to underlying hematological malignancy. This contrasts with the work of McCarty et al., who presented a 29% rate of fungal cellulitis of the orbit from sinusitis in children with neutropenia and fever [15]. This difference might be because our studied population was adults who could express their discomfort accurately. Patients in McCarty's work were aged from 4 months to 15 years. The difficulty of examination and subjective complaints may interfere with initial detection. Besides, due to the national health insurance in Taiwan, it was much easier and affordable for high-cost image study such as CT, MRI, and even endoscopy, which might also increase early diagnosis of sinusitis and prevent further disease progression.

Sinusitis is mostly presented as nasal discharge, nasal congestion, cheek pain or numbness, and a smelly odor. However, in our case series, less than half of the patients complained about these typical sinusitis symptoms. We supposed this was due to the following possibilities: first, most patients were under an immunocompromised status (e.g., poorly controlled DM or hematological malignancy) causing the inflammatory reaction to be less severe. Second, patients with DM may have neuropathy which caused less cheek pain or numbness. Third, some patients were not fully conscious prior to admission, so their family members were unable to properly illustrate the initial subjective symptoms. On the other hand, ptosis, facial or orbital swelling, and EOM limitation were objective, allowing for practitioners to well document. Several previously published articles have described fungal orbital cellulitis symptoms. Bhansali et al. had documented 35 patients who had been diagnosed with rhino-orbital-cerebral mucormycosis (ROCM); in which 89% of the patients complained about external ophthalmoplegia, proptosis (83%), visual loss (80%), and chemosis (74%) [10]. These were similar to our findings. Another article from India studied 34 ROCM patients, where loss of vision was documented in 67.6% of the patients and central retinal artery occlusion (CRAO) occurred in 50% [13]. In our study, eight of the nine orbital-involved patients had complained about decreased visual acuity, and all showed intraconal infiltration on orbital image and some had optic nerve swelling.

Patients without orbital involvement experienced a higher prevalence of fever (100% compared to 33.3% in orbital cellulitis). Fever may be associated with hematological malignancy, neutropenia status, or other opportunistic coinfections in this group. On the other hand, five patients in the fungal orbital cellulitis group who experienced conscious change at presentation or at their beginning days of hospitalization died due to progressive infection. We assumed that conscious disturbance indicated progressive infection such as sepsis status or central nervous system involvement.

Orbital cellulitis mostly occurs when infection spreads from the paranasal sinuses, and most commonly originates from the ethmoid sinus through the thin lamina papyracea of the medial orbital wall. There are two ways for it to spread: direct invasion through osteitis or through infective emboli along the ethmoid vein [8, 10, 16]. We found a significantly higher involvement rate for frontal, ethmoid, and sphenoid sinus infection in patients with orbital disease. The sphenoid sinus opens into the sphenoethmoidal recess of the nasal cavity, while the frontal sinus drains into the middle meatus. The anterior and posterior ethmoidal cells drain into the middle and superior meatuses, respectively. Most orbital infection presented as medial and inferior orbital infiltration in our study. Five of these 9 orbital-involved patients had subperiosteal abscess over medial or medial upper orbit, and 3 of the 5 patients had further encephalitis or even brain abscess. Medial rectus muscle and inferior rectus muscle engorgement were seen in 6 of these 9 patients. Also, according to Bhansali et al., the ethmoid sinus (86%) was most frequently involved in ROCM patients [10]. We can infer that further orbital or even brain involvement should be a concern when a CT or MRI reveals ethmoid, sphenoid, or frontal sinusitis, even if the patient presented no suspicious symptoms.

In our series, *Rhizopus* infection was involved in 4 of 9 orbital disease patients, whereas it was only seen in 1 no orbital involvement patient. *Aspergillus* was highly associated with orbital sparing patients, as it was seen in 9 of 12 patients. These pathogen differences were also documented in another article. Trief et al. found mucormycosis accounted for 79% of orbital disease and *Aspergillus* accounted for 60% of orbit sparing disease in their retrospective study [17]. Mucormycosis is caused by order Mucorales, and *Rhizopus oryzae* is the most common organism, accounting for 70–90% orbital mucormycosis according to the literature. *Aspergillus* is a spore-forming, septate filamentous mold. *Aspergillus flavus* and *Aspergillus fumigatus* most commonly affect sinuses and orbit [18].

Despite multiantifungal medication use, AIFRS and rhino-orbital infection remain hazardous conditions for clinical practitioners. The disease displays characteristics of rapid progression and high mortality rate. Some consensuses have been made on treatment: (1) initiating antifungal medication, (2) debridement of necrotic tissue, and (3) reducing the immunosuppression conditions [19]. In our series, all AIFRS patients received amphotericin B or liposomal amphotericin B for *Mucor*, *Rhizopus,* or *Rhizomucor* and voriconazole for *Aspergillus*, while some patients received posaconazole due to side effects of amphotericin B or preparing for discharge. The systemic antifungal agents were similar to those in other articles. Amongst the 9 patients diagnosed with fungal orbital cellulitis, all had received ESS, with 6 of them receiving more than one ESS. Evisceration, incision, and drainage of brain abscess, anterior orbitotomy, and intravitreal injection of amphotericin B were performed on 4 different patients, all of whom died during hospitalization. The previous literature has emphasized early aggressive debridement, with exenteration being a possible choice, although that is still under debate. Many authors have discussed mortality rates and exenteration, with Hirabayashi et al. finding no differences in the mortality rate between subjects who either did or did not have their orbit exenterated [20]. Besides, Athavale et al. reported consecutive 6 patients with sino-orbital fungal disease preferably be treated conservatively, without orbital exenteration [21]. Trief et al. have suggested that exenteration should be considered for individual patients [17]. None of our patients received exenteration, although one did receive evisceration. This may be related to difficult decision making by family members, while also being associated with traditional Taiwanese religion. Mortality rates for invasive fungal rhinosinusitis range from 6 to 68% [19], and a review article illustrated that mortality rate for ROCM was approximately 50% with amphotericin B and surgical debridement [18]. Trief et al. reported a mortality rate of 78.6% for orbital disease and 20% for sinus-only disease [17]. The result of our case series for orbital disease showed a 55.6% mortality rate, which was similar to that found in the previous literature. Additionally, the mortality rate for the orbital sparing group in our study was 58.3%; however, all these patients later died due to hematological malignancy.

Comparing the parameters of the expired and alive patients in the orbital-involved group, only the consciousness change revealed significance. We suggest that this may indicate CNS involvement or a severe sepsis-related condition. Three of the 5 expired patients had brain edema or abscess, while one had intracranial hemorrhage due to thrombocytopenia caused by sepsis. Besides, all these patients had comorbidity of diabetes. Mostly, clinicians regard that patients with hematological malignancy have poor immunity. However, our research showed that the prognosis of infection in diabetic patients is much poor. The reasons are presumed to be as follows: (1) diabetic patients are treated in outpatient clinic, and most patients would not visit medical centers when feeling discomfort. (2) Patients with malignancy are very alert to the changes in their conditions, and they often return to the clinic or be hospitalized for cancer treatment, and medical care is highly accessible for them.

To our knowledge, this retrospective study was the first one that analyzed the possible risk factors of orbital infection among AIFRS patients in Asia. However, there were some limitations to this study. First, it was of a retrospective nature and the data were collected from chart reviews. Most patients were admitted to the internal medicine ward, where either the otorhinolaryngologist or ophthalmologist was consulted when nasal or orbital symptoms were complained about. Thus, the initial presentation offered a limited view. Second, due to the large differences among the comorbidities in these patients, the treatment plan was highly variable, with no treatment guidelines or protocols having been established in our hospital. Lastly, acute invasive fungal rhinosinusitis is a rare disease and our study sample was small and, therefore, may not represent all features and disease course.

In conclusion, acute invasive fungal rhinosinusitis is a devastating disease with rapid progression and a high mortality rate. We found that patients with diabetic mellitus experienced a higher possibility of orbital infection. Additionally, frontal, sphenoid, and ethmoid sinuses involvement had a positive correlation with orbital involvement. Rhizopus was the most common pathogen in orbital involvement disease. Infection-related death was much higher when the orbit was involved, and patients with conscious disturbance had higher possibility of death. Practitioners should be aware of the abovementioned factors and make efforts on early detection and prevention of orbital or even cerebral involvement in acute invasive fungal rhinosinusitis.

## Figures and Tables

**Table 1:** Comparison of patients' characteristics, symptoms and signs, culture reports, and image findings between the orbital-involved and sparing group in patients diagnosed with AIFRS (DM: diabetes mellitus; EOM: extraocular movement).

	Orbital involvement (*n* = 9)	Orbital sparing (*n* = 12)	Total (*n* = 21)	*p* value
*Basic information*				
Male	6 (66.7%)	7 (58.3%)	13 (61.9%)	1.000
Age, years^†^	64.0 (56.5–71.5)	55.0 (37.5–67.8)	58.0 (47.0–69.5)	0.109
Onset days^†^	14.0 (8.5–17.0)	13.0 (2.8–19.3)	14.0 (6.5–18.5)	0.668

*Comorbidity*				
DM	9 (100.0%)	2 (16.7%)	10 (47.6%)	<0.001^*∗∗*^
Hypertension	7 (77.8%)	1 (8.3%)	7 (33.3%)	0.002^*∗∗*^
Renal insufficiency	4 (44.4%)	0 (0.0%)	4 (19.0%)	0.021^*∗*^
Systemic steroid use	1 (11.1%)	1 (8.3%)	2 (9.5%)	1.000
Malignancy solid tumor	0 (0.0%)	2 (16.7%)	2 (9.5%)	0.486
Malignancy hematology	1 (11.1%)	8 (66.7%)	9 (42.9%)	0.024^*∗*^

*Symptoms and signs*				
Nasal obstruction	1 (11.1%)	4 (33.3%)	5 (23.8%)	0.338
Nasal discharge	4 (44.4%)	3 (25.0%)	7 (33.3%)	0.397
Cheek pain	4 (44.4%)	4 (33.3%)	8 (38.1%)	0.673
Facial swelling	1 (11.1%)	2 (16.7%)	3 (14.3%)	1.000
Ptosis	4 (44.4%)	1 (8.3%)	5 (23.8%)	0.119
Orbital pain	6 (66.7%)	2 (16.7%)	8 (38.1%)	0.032^*∗*^
Orbital swelling	4 (44.4%)	1 (8.3%)	5 (23.8%)	0.119
Decreased visual acuity	8 (88.9%)	0 (0.0%)	8 (40.0%)	<0.001^*∗∗*^
EOM limitation	7 (77.8%)	0 (0.0%)	7 (35.0%)	<0.001^*∗∗*^
Fever	3 (33.3%)	12 (100.0%)	15 (71.4%)	0.002^*∗∗*^
Headache	4 (44.4%)	1 (8.3%)	5 (23.8%)	0.119
Conscious disturbance	5 (55.6%)	1 (8.3%)	6 (28.6%)	0.046^*∗*^

*Fungal culture report*				
*Mucor*	2 (22.2%)	1 (8.3%)	3 (14.3%)	0.553
*Rhizomucor*	1 (11.1%)	0 (0.0%)	1 (4.8%)	0.429
*Rhizopus*	4 (44.4%)	1 (8.3%)	5 (23.8%)	0.119
*Aspergillus flavus*	3 (33.3%)	9 (75.0%)	12 (57.1%)	0.087
*Fusarium*	0 (0.0%)	1 (8.3%)	1 (4.8%)	1.000

*Sinus involvement on CT or MRI*				
Frontal sinus	6 (66.7%)	1 (8.3%)	7 (33.3%)	0.016^*∗*^
Ethmoid sinus	9 (100.0%)	4 (33.3%)	13 (61.9%)	0.005^*∗∗*^
Sphenoid sinus	8 (88.9%)	2 (16.7%)	10 (47.6%)	0.002^*∗∗*^
Maxillary sinus	8 (88.9%)	8 (66.7%)	16 (76.2%)	0.338

Chi-square test or Fisher's exact test. Mann–Whitney *U* test, median (IQR). ^*∗*^*p* < 0.05, ^*∗∗*^*p* < 0.01.

**Table 2:** The comorbidities, symptoms and signs, and image findings of alive or expired patients with fungal orbital infection related to AIFRS (DM: diabetes mellitus; EOM: extraocular movement).

	Alive (*n* = 4)	Dead (*n* = 5)	*p* value
*Basic information*			
Male	3 (75.0%)	3 (60.0%)	1.000
Age, years	62.0 (48.0–73.0)	67.0 (56.5–71.5)	0.806
Onset days	10.5 (7.0–14.0)	14.0 (11.0–20.5)	0.211

*Comorbidity*			
DM	4 (100.0%)	5 (100.0%)	—
Hypertension	2 (50.0%)	5 (100.0%)	0.167
Renal insufficiency	2 (50.0%)	2 (40.0%)	1.000
Malignancy hematology	0 (0.0%)	1 (20.0%)	1.000
Malignancy solid tumor	0 (0.0%)	0 (0.0%)	—
Systemic steroid use	1 (25.0%)	0 (0.0%)	0.444
HbA1C (%)	10.7 (9.28–13.98)	10.0 (8.0–14.0)	0.480

*Symptoms and signs*			
Fever	1 (25.0%)	3 (60.0%)	0.524
Headache	2 (50.0%)	2 (40.0%)	1.000
Nasal obstruction	1 (25.0%)	0 (0.0%)	0.444
Nasal discharge	2 (50.0%)	2 (40.0%)	1.000
Cheek pain	2 (50.0%)	3 (60.0%)	1.000
Facial swelling	0 (0.0%)	1 (20.0%)	1.000
Orbital pain	2 (50.0%)	4 (80.0%)	0.524
Orbital swelling	1 (25.0%)	3 (60.0%)	0.524
Ptosis	1 (25.0%)	3 (60.0%)	0.524
Decreased visual acuity	3 (75.0%)	5 (100.0%)	0.444
EOM limitation	3 (75.0%)	4 (80.0%)	1.000
Conscious disturbance	0 (0.0%)	5 (100.0%)	0.008^*∗∗*^

*Image findings*			
Orbital subperiosteal abscess	1 (25.0%)	4 (80.0%)	0.206
Orbital intraconal infiltration	3 (75.0%)	5 (100.0%)	0.444
Extraocular muscles swelling	2 (50.0%)	4 (80.0%)	0.524
Optic nerve swelling	1 (25.0%)	3 (60.0%)	0.524
Encephalitis (brain edema and brain abscess)	0 (0.0%)	3 (60.0%)	0.167

Fisher's exact test. ^*∗∗*^*p* < 0.01.

## Data Availability

The data of this study are available from the corresponding author upon request.
